# Does dehydration prior to primary total joint arthroplasty increase risk of perioperative complications?

**DOI:** 10.1186/s42836-021-00090-8

**Published:** 2021-10-04

**Authors:** Harold G. Moore, Marissa A. Justen, David S. Kirwin, Patrick J. Burroughs, Lee E. Rubin, Jonathan N. Grauer

**Affiliations:** 1 Weill Cornell Medical College, New York, NY USA; 2 Yale School of Medicine, New Haven, CT USA; 3 Department of Orthopaedics and Rehabilitation, Yale School of Medicine, 47 College Street, New Haven, CT 06511 USA

**Keywords:** Total knee arthroplasty, Total hip arthroplasty, Dehydration, Hydration, Transfusion

## Abstract

**Background:**

Prior studies have suggested that preoperative dehydration increases odds of perioperative complications in several areas of orthopedic surgery. This study aimed to evaluate whether preoperative hydration status is associated with the incidence of short-term complications after primary total joint arthroplasty.

**Methods:**

The 2012–2019 National Surgical Quality Improvement Program (NSQIP) database was used to explore the relationship between preoperative dehydration (ratio of preoperative BUN divided by preoperative Creatinine (BUN/Creatinine) > 20) and perioperative outcomes of total hip (THA) and total knee arthroplasty (TKA) patients. Univariate comparisons and multivariate regression analyses were conducted to identify specific complications that occurred more often in patients with preoperative dehydration.

**Results:**

Of 188,629 THA and 332,485 TKA patients, 46.3 and 47.0% had preoperative dehydration, respectively. After controlling for demographics and comorbidities, dehydrated THA patients were no more likely to experience a complication compared to their non-dehydrated counterparts (relative risk [RR] = 0.97, 99.7% Confidence Interval [CI]: 0.92–1.03, *P* = 0.138) nor increased risk of blood transfusion (RR = 1.02, CI = 0.96–1.08, *P* = 0.408). Similar to THA patients, dehydrated TKA patients were not more likely to have a complication after surgery (RR = 0.97, CI = 0.92–1.03, *P* = 0.138) and were at no greater risk of transfusion (RR = 1.02, CI = 0.96–1.07, *P* = 0.408). A sub-analysis covering only patients with BUN and Cr values determined within 24 h after surgery was performed and similarly found no significant increase in perioperative complications or transfusion.

**Conclusion:**

Overall, preoperative dehydration in patients undergoing THA/TKA did not appear to increase risk of transfusion or other perioperative complications. Further research is needed to characterize the role of hydration prior to elective total joint arthroplasty.

**Supplementary Information:**

The online version contains supplementary material available at 10.1186/s42836-021-00090-8.

## Introduction

There is an increasing emphasis in total joint arthroplasty on optimizing the patient prior to surgery to reduce risk of postoperative complications and expedite recovery. Attention is often paid to exercise tolerance [1], optimizing management of medical comorbidities, and performing routine laboratory testing to ensure there are no gross electrolyte [2], nutritional [3], or hematologic abnormalities [4, 5]. One factor that has garnered increased attention in several areas of orthopedics is preoperative hydration status [6].

Patients are often dehydrated prior to surgery and can require fluid not only to replace intraoperative and postoperative fluid losses but also to assist in replenishing fluid deficits present prior to surgery. Nausea and vomiting are frequent side effects of the postoperative narcotic regimen [7] and can result in further fluid loss, and patients are often restricted in their oral intake prior to surgery for anesthetic safety similar to how patient intake is restricted prior to drawing blood for routine laboratory tests [8]. Several studies examining the role of oral and intravenous preoperative hydration found that such protocols improved postoperative recovery with less nausea and vomiting [9, 10].

Hydration status can be inferred in a number of ways, including clinical signs such as skin turgor, flattening of neck veins, and more quantifiable blood pressure, heart rate, and urine specific gravity [11]. Another widely adopted metric for assessing hydration status is the Blood Urea Nitrogen (BUN)/Creatinine (Cr) ratio. In the literature, thresholds for the dehydrated state range from a ratio greater than 15 to a ratio greater than 25, but the threshold is most commonly set at a ratio greater than 20 [12, 13]. However, the relationship between preoperative hydration status and postoperative complications in total joint arthroplasty is largely unexplored.

Total joint arthroplasty is one of the most common orthopedic procedures performed in the United States and is increasing in frequency every year as the population ages, with the combined volume of primary total hip and total hip procedures expected to approach 2 million annually by 2030 [14]. Given the susceptibility of elder patients to changes in hydration status and the high prevalence of dehydration in this age group [15], the authors hypothesized that preoperative hydration status in TJA may increase the risk of complications within the perioperative period, including transfusion, infection, acute kidney injury, and hospital readmission [16].

For the current study, a large national surgical database was used to examine whether preoperative patient hydration status, as defined by BUN/Cr ratio, is associated with increased incidence of 30-day postoperative complications.

## Materials and methods

### Data source

The American College of Surgeon’s NSQIP Database is a prospective, risk-adjusted database administered by the American College of Surgeons that collects data on patient demographics and comorbidities, perioperative characteristics and postoperative complications through the 30^th^ postoperative day regardless of discharge destination for over 700 participating institutions in the United States. Clinical reviewers employed by each participating hospital gather data directly from patient charts and input data into the database, and data are regularly audited for accuracy. Our institutional review board has granted exemption to studies using this de-identified database.

### Patient population

**Fig. 1:**
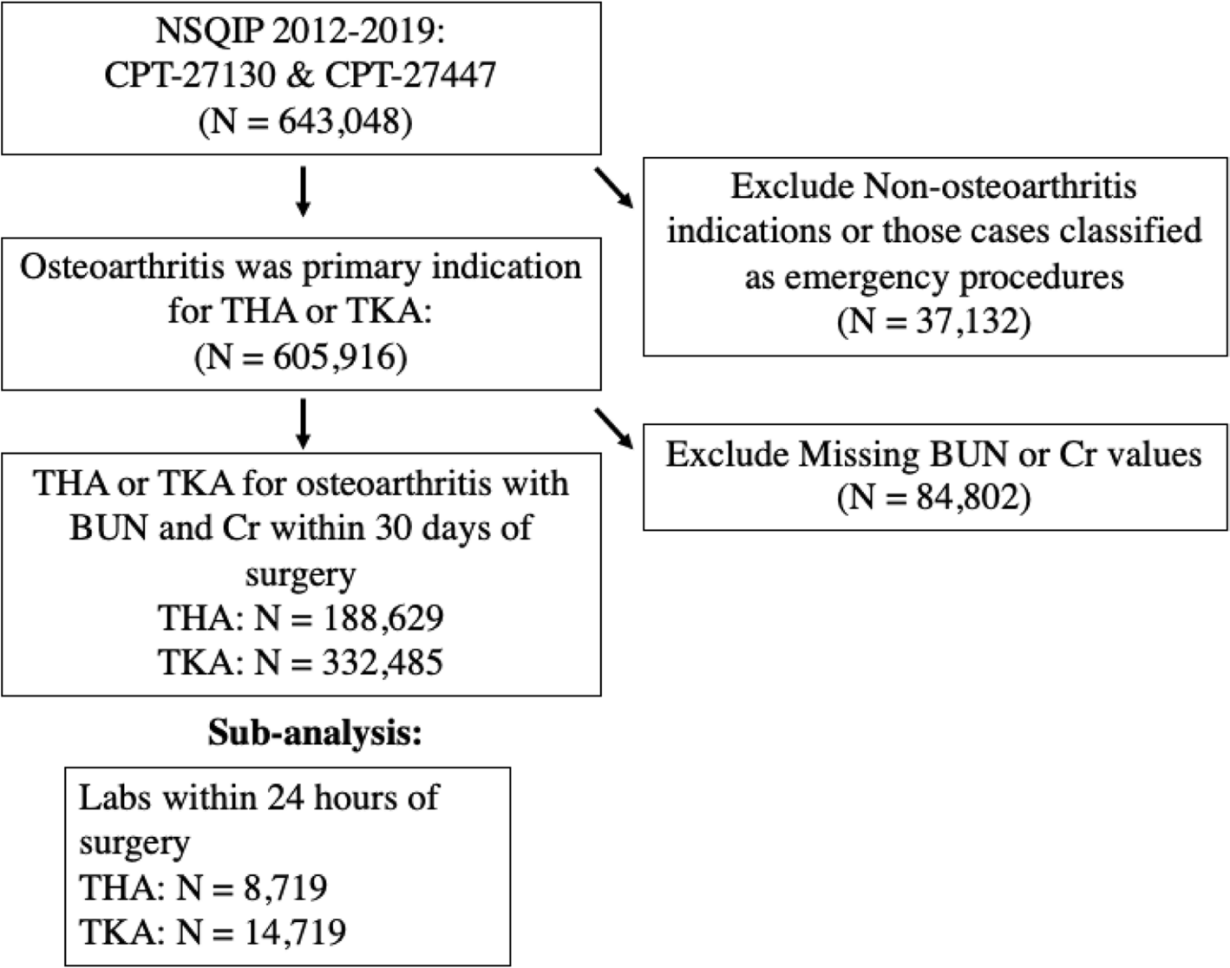
Flow diagram representing arrival at overall patient population as well as the population used for supplemental sub-analysis of those patients with BUN and creatinine labs drawn within 24 h of surgery

Patients undergoing elective total hip arthroplasty (THA) and total knee arthroplasty (TKA) from the years 2012 to 2019 were identified using the Current Procedural Terminology (CPT) Code 27477 and 27130, respectively. Only those patients with complete data for all preoperative and postoperative variables were included. Patients who underwent surgery for diagnoses other than osteoarthritis (such as for infections, neoplasia, or revision as identified by their International Classification of Disease code) were also excluded from the study. Patients with chronic renal failure or who were on dialysis prior to surgery were also excluded because BUN and creatinine levels were elevated and were a poor indicator of hydration status in these populations [17]. Figure 1 illustrates how the ultimate study population was determined.

Preoperative characteristics were obtained directly from NSQIP and included age, sex, height, weight, functional status, American Society of Anesthesiologists (ASA) score, diabetes status, smoking status, and congestive heart failure (CHF). Body mass index (BMI) was calculated using height and weight data (kg/m^2^), which are chart-abstracted and can be either objective measures or self-reported measures, depending on the practice of individual NSQIP member institutions. ASA class was used as a simplified measure of patient comorbidity status and has been demonstrated to correlate strongly with other comorbidity indices [18, 19].

### Perioperative complications

NSQIP follows up individual complications in 30-day postoperative period, regardless of discharge destination. The individual complications included transfusion, death, urinary tract infection (UTI), pneumonia, return to OR, wound-related complications, thromboembolic events, sepsis and septic shock, cardiac complications, renal complications, and cerebrovascular accident. In the current study, pulmonary embolism and deep vein thrombosis were categorized as “thromboembolic events,” while superficial surgical site infection, deep wound infection, wound dehiscence, and organ space infection were categorized as “wound-related complications.” Blood transfusion was noted if blood was given during surgery or within the first 72 h postoperatively.

Postoperative length of stay (LOS) and readmission were also directly reported in the NSQIP database. LOS is defined as the number of days from the operation date until discharge (with maximum of 30 days). Extended LOS was defined as any LOS greater than 1 standard deviation above the mean for all cases (3 days for both THA and TKA). Readmission was noted if there was an admission for any reason occurring within the 30-day postoperative period.

In the NSQIP database, the laboratory values and the date the laboratory values were drawn are recorded. The blood BUN and Cr values were always recorded on the same date as both are drawn together as part of the basic metabolic panel. BUN/Cr values in NSQIP were recorded closest to surgery with no limit to the number of days prior to surgery that most recent value was recorded. A sub-analysis was additionally performed specifically examining patients within 24 and 72 h prior to surgery to evaluate possible bias between time and surgery and laboratory draws (Supplemental Tables 1, 2, 3 and 4).

### Statistical analysis

All statistical analyses were completed using Stata version 15. To begin with, patients who underwent THA and TKA were divided into two categories: BUN/Cr ≤ 20 and BUN/Cr > 20. Preoperative demographics and comorbidities between the 2 groups were compared in a univariate fashion using Chi-Squared test with significance set at *P* < 0.05.

Next, multivariate Poisson regression with robust error variance was used to compare the relative risk of adverse outcomes that occurred within the 30-day postoperative period. Multivariate regressions were controlled for preoperative characteristics that were found to be significant in the univariate comparisons. Because 15 outcomes were examined, Bonferroni’s correction was used to denote significance at *P* < 0.003. Accordingly, 99.7% confidence intervals were reported.

## Results

### Total hip arthroplasty cohorts

A total of 188,629 THA patients met inclusion/exclusion criteria. Of these patients, 87,337 (46.3%) were dehydrated based on preoperative laboratory studies. These patients who fit the definition for having preoperative dehydration were: older, more likely to be female, have lower BMI, have a preoperative dependent functional status, have a higher ASA, have diabetes, and have congestive heart failure (CHF). Of note, those patients that met the criteria for dehydration had a lower prevalence of tobacco use (Table 1). When only those patients with BUN and Cr values within 24 h of surgery were evaluated, they had similar findings but did not have increased prevalence of dependent functional status or CHF (Supplemental Table 1).

**Table 1:** Demographics and comorbidities for patients undergoing primary total hip arthroplasty

**Demographics and Comorbidities**	**Hydration Status**				***P***^**a**^
	**Non-Dehydrated**		**Dehydrated**		
	***n* = 101,292**		***n* = 87,337**		
Age (years) mean ± SD	63.9 ± 11.0		67.7 ± 10.2		** < 0.001**
Gender					** < 0.001**
Female	45,210	44.6%	58,657	67.2%	
BMI (kg/m^2^)					** < 0.001**
< 18	333	0.3%	492	0.6%	
18–25	16,522	16.3%	17,773	20.4%	
25–30	34,058	33.6%	28,490	32.6%	
30–35	28,208	27.9%	21,806	25.0%	
35–40	14,546	14.4%	12,053	13.8%	
> 40	7625	7.5%	6723	7.7%	
Functional status prior to surgery					** < 0.001**
Independent	99,871	98.6%	85.864	98.3%	
Dependent	1421	1.4%	1473	1.7%	
ASA					** < 0.001**
1	3775	3.7%	2822	3.2%	
2	53,429	52.8%	46,766	53.6%	
3	42,353	41.8%	36,260	41.5%	
≥ 4	1735	1.7%	1489	1.7%	
Diabetes mellitus					** < 0.001**
No diabetes mellitus	89,009	87.8%	76,018	87.0%	
Non-insulin-dependent diabetes mellitus	9488	9.4%	8758	10.0%	
Insulin-dependent diabetes mellitus	2795	2.8%	2561	3.0%	
Smoker					** < 0.001**
No	86,850	85.7%	79,577	91.1%	
Yes	14,442	14.3%	7760	8.9%	
Congestive heart failure					**0.007**
No	100,996	99.7%	87,020	99.6%	
Yes	296	0.3%	317	0.4%	

Bold fonts indicate statistical significance

^a^Statistical significance set at *P* < 0.05

On multivariate analysis, dehydrated patients undergoing THA were not more likely to have a complication (relative risk [RR] = 0.96, 99.7% confidence interval [CI]: 0.89–1.04, *P* = 0.135) and did not have an increased postoperative requirement for blood transfusion, (RR = 1.02, CI: 0.96–1.08, *P* = 0.408) (Table 2, Fig. 1). Similarly, the sub-analysis of patients having laboratory tests within 24 h of surgery also showed that no increased risk of complications was observed (Supplemental Table 2). However, the risk of readmission within 30 days of surgery was significantly lower (RR = 0.92, CI = 0.85–0.99, *P* = 0.001), with the absolute readmission rate being roughly 3% in both cohorts.

**Table 2:** Relative risk of complications in the 30-day postoperative period for patients undergoing primary total hip arthroplasty

**Total**	**RR**^**a**^	**99.7% CI**	***P***^**b**^
Any Complication	0.96	0.89–1.04	0.135
Extended length of stay (> 3 days)	0.98	0.94–1.02	0.164
Blood transfusion	1.02	0.96–1.08	0.408
**Readmission**	**0.92**	**0.85**–**0.99**	**0.001**
Death	0.90	0.60–1.35	0.437
Urinary tract infection	1.00	0.86–1.17	0.970
Pneumonia	0.81	0.62–1.06	0.021
Return to the operating room	0.98	0.88–1.08	0.507
Wound complications	0.95	0.83–1.09	0.269
Thromboembolic complications^c^	0.94	0.77–1.13	0.303
Sepsis/septic shock	0.88	0.66–1.17	0.189
Respiratory complications	0.76	0.52–1.12	0.035
Cardiac complications	1.06	0.81–1.38	0.527
Renal complications	0.80	0.53–1.21	0.110
Cerebrovascular accident	0.85	0.54–1.34	0.293

^a^Poisson regressions with robust error variance were used to compare these variables. Adjusted for age, gender, BMI, functional status, ASA class, diabetes, smoking status, and congestive heart failure

^b^Bold fonts indicate statistical significance (significant at *P* < 0.003 due to Bonferroni correction for multiple comparisons). *RR* Relative risk, *CI* Confidence interval (99.7% CI due to Bonferroni)

^c^Includes deep vein thrombosis and pulmonary embolism

### Total knee arthroplasty cohorts

**Fig. 2:**
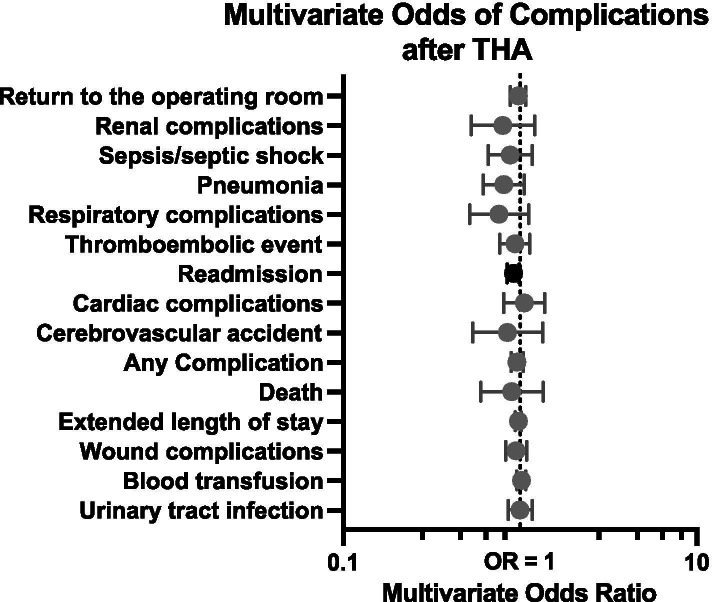
Relative risk of complications that occurred anytime during the 30-day postoperative period following total hip arthroplasty in non-dehydrated *vs*. dehydrated patients. Dark shading indicates statistical significance

A total of 332,485 TKA patients met inclusion/exclusion criteria. Of these patients, 156,113 (47.0%) were dehydrated based on preoperative laboratory studies. These patients who fit the definition for having preoperative dehydration were: older, more likely to be female, have lower BMI, higher ASA, be diabetic, and less likely to smoke (Table 3, Fig. 2). The dehydrated TKA patients were similar with respect to functional status and CHF. In the stricter sub-analysis of BUN/Creatinine values drawn within 24 h of surgery, the same similarities and differences in demographic and comorbidity factors were evident (Supplemental Table 3).

**Table 3:** Demographics and comorbidities for patients undergoing primary total knee arthroplasty

**Demographics and Comorbidities**	**Hydration Status**				***P*-value**^**a**^
	**Non-Dehydrated**		**Dehydrated**		
	***N* = 176,372**		***N* = 156,113**		
Age (years) mean ± SD	65.9 ± 9.5		68.2 ± 9.0		** < 0.001**
Sex					** < 0.001**
Female	14,309	8.1%	16,015	10.3%	
BMI (kg/m^2^)					** < 0.001**
< 18	181	0.1%	221	0.1%	
18–25	14,309	8.1%	16.015	10.3%	
25–30	47,112	26.7%	41,306	26.5%	
30–35	52,210	29.8%	44,598	28.6%	
35–40	25,971	20.4%	30,836	19.8%	
> 40	26,289	14.9%	23,137	14.8%	
Functional status prior to surgery					0.528
Independent	174,574	99.9%	154,487	99.0%	
Dependent	1798	1.0%	1626	1.0%	
ASA					** < 0.001**
1	3030	1.7%	2547	1.6%	
2	82,401	46.7%	76,620	49.1%	
3	87,695	49.7%	74,604	47.8%	
≥ 4	3246	1.8%	2342	1.5%	
Diabetes mellitus					**0.032**
No diabetes mellitus	143,737	81.5%	127,171	81.5%	
Non-insulin-dependent diabetes mellitus	24,634	14.0%	22,107	14.0%	
Insulin-dependent diabetes mellitus	8001	4.5%	6835	4.4%	
Smoker					** < 0.001**
No	158,498	89.9%	147,051	94.2%	
Yes	17,874	10.1%	9062	5.8%	
Congestive heart failure					0.707
No	175,794	99.7%	155,613	99.7%	
Yes	578	0.3%	500	0.3%	

Bold fonts indicate statistical significance.

^a^Statistical significance set at *P* < 0.05

**Fig. 3:**
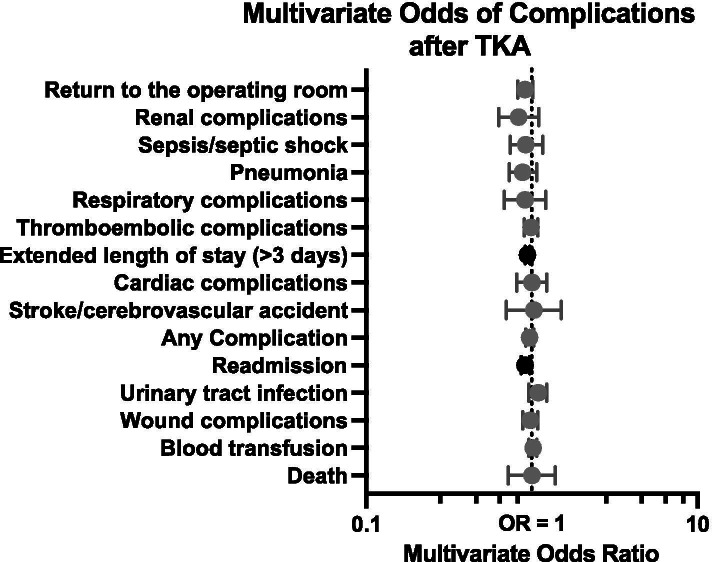
Relative risk of complications that occurred anytime during the 30-day postoperative period following total knee arthroplasty in non-dehydrated *vs*. dehydrated patients. Dark shading indicates statistical significance

Like THA patients, dehydrated TKA patients were not more likely to have a complication after surgery (RR = 0.97, CI: 0.92–1.03, *P* = 0.138). Dehydrated TKA patients did not experience increased risk of perioperative blood transfusion either (RR = 1.02, CI: 0.96–1.07, *P* < 0.001). Most other complications did not have an increased risk of complications (Table 4, Fig. 3). Lastly, as found in THA patients, TKA patients who met laboratory criteria for dehydration had significantly reduced risk of readmission (RR = 0.91, CI: 0.86–0.97, *P* =  < 0.001), or extended length of stay (RR = 0.94, CI: 0.91–0.97, *P* =  < 0.001). In the sub-analysis, no significant increase in complications was noted (Supplemental Table 4).

**Table 4:** Relative risk of complications in the 30-day postoperative period for patients undergoing primary total knee arthroplasty

**Adverse Event**	**RR**^**a**^	**99.7% CI**	***P*-value**^**b**^
Any Complications	0.97	0.92–1.03	0.138
Urinary tract infection	1.09	0.96–1.23	0.040
Blood transfusion	1.02	0.96–1.07	0.408
**Extended length of stay (> 3 days)**	**0.94**	**0.91**–**0.97**	** < 0.001**
**Readmission**	**0.91**	**0.86**–**0.97**	** < 0.001**
Death	1.00	0.72–1.39	0.973
Pneumonia	0.88	0.73–1.07	0.059
Return to the operating room	0.91	0.82–1.01	0.007
Wound complications	0.98	0.88–1.09	0.586
Thromboembolic complications^c^	0.99	0.90–1.09	0.825
Sepsis/septic shock	0.92	0.74–1.16	0.303
Respiratory complications	0.91	0.68–1.21	0.322
Cardiac complications	1.00	0.81–1.23	0.993
Renal complications	0.83	0.63–1.10	0.047
Stroke/cerebrovascular accident	1.03	0.70–1.51	0.805

^a^Poisson regressions with robust error variance were used to compare these variables. Adjusted for age, gender, BMI, ASA class, diabetes, and smoking status

^b^Bold fonts indicate statistical significance (significant at *P* < 0.003 due to Bonferroni correction for multiple comparisons). *RR* Relative risk, *CI* Confidence interval (99.70% CI due to Bonferroni)

^c^Includes deep vein thrombosis and pulmonary embolism

## Discussion

Optimizing patients prior to elective primary total joint arthroplasty is key to ensuring rapid postoperative recovery. While much attention has been directed toward medical management of recognized comorbid conditions, only recently has more attention been given to preoperative hydration status. The current study aimed to examine the association between preoperative dehydration and postoperative outcomes after THA and TKA.

Dehydration has long been established as a factor in recovery after several orthopedic surgeries [20, 21]. One small prospective study of 45 patients undergoing surgery for hip fracture used urine specific gravity upon arrival to the operating theater to quantify preoperative dehydration and found a quadrupled postoperative complication rate in the dehydrated group [22]. Another small study looking at only patients over 65 undergoing several different types of orthopedic surgery found similar results, including increased rates of readmission and overall mortality at 30 days [2].

In general, the association of preoperative hydration status with specific preoperative outcomes of THA and TKA have largely been unexamined. However, the role of hydration status in orthopedic surgical outcomes was more broadly assessed by both Ylinenvaara *et al*. and Chan *et al*. [12, 22]. Ylinenvaara *et al*. demonstrated that patients who were dehydrated were four times at the risk of experiencing an adverse postoperative outcome, and, more specifically, were more likely to have confusion, arterial desaturation and cardiovascular events. These conclusions were not supported by our findings. Chan *et al*., after examining the records of 216 patients, concluded that patients who had preoperative dehydration status were more likely to experience hematologic or gastrointestinal postoperative complications which is consistent with our finding of increased need for blood transfusion in both THA and TKA patients who were dehydrated prior to surgery [12]. The main difference between our study and the studies previously mentioned is that previous studies examined orthopedic surgeries more broadly, rather than patients specifically undergoing elective THA and TKA. Moreover, the mean age of study populations in Chan *et al*. [12] (81 years), and Ylinevaara *et al*. [22] (78 years), was not accounted for using multivariate analysis as it was in the present study. Accordingly, age and procedure type could have contributed to the differences these studies observed.

Based on univariate analyses, the current study found that both THA and TKA patients with preoperative dehydration were older, mostly female, and had a higher medical comorbidity burden than non-dehydrated patients. The above-noted findings align with previous literature that has demonstrated that dehydrated patients were, on average, older and more likely to be female than non-dehydrated patients [23]. Interestingly, those patients who were smokers were disproportionately more represented in the non-dehydrated group, a finding that may be partly explained by observations of altered creatine clearance in smokers [24, 25]. Based on multivariate analyses, the current study found that patients with preoperative dehydration undergoing both THA and TKA experienced reduced risk of postoperative complications within 30 days of surgery. For TKA in particular, they had slightly but significantly reduced risk of extended length of stay and were not more likely to require a blood transfusion. While the adjusted increase in relative risk was small, with large and increasing volume of THAs and TKAs being performed globally [26], this small difference at scale can be clinically significant.

For TKA and THA patients, the current study found that preoperative dehydration did not result in an appreciable elevation in the risk of complications. Of note, the sub-analysis conducted for patients within 24 h of surgery had a less statistical power and did not find any significant increase in complications, as patients undergoing laboratory testing within 24 h of surgery comprised only 4.5% of the overall study population. This should be taken into account when considering the generalizability of the results of the sub-analysis and highlights the need to conduct more regimented studies drawn at pre-defined intervals prior to surgery.

Recently, novel multi-modal approaches have been developed to try to reduce transfusions after total joint arthroplasty by optimizing preoperative hemoglobin, reducing perioperative blood loss, and imposing strict transfusion requirements. In one study of 1010 patients, the transfusion rate was reduced from 18 to 1.4% by applying these measures in addition to optimizing hydration prior to surgery [27]. In our study population, the transfusion rate was 5.6 and 3.4% in THA and TKA respectively, suggesting that further reduction is possible. One factor that might explain the high transfusion rates compared to contemporary rates is use of tranexamic acid (TXA). TXA’s use was not popularized in total joint arthroplasty in the most of the years of the study but is likely represented to a more appreciable degree during the last several years in the study [28].

More specific to total joint arthroplasties, a study by Mukand *et al*. [29] in 2003 examined the effects of dehydration on in-patient rehabilitation of patients who underwent THA, TKA, or had a hip fracture by using orthostasis and azotemia as metrics of dehydration. However, they found that, contrary to the present study, dehydrated patients were at a higher risk of having an extended length of hospital stay than non-dehydrated patients [29]. The inclusion of hip fracture patients may have confounded their analysis, as there is an increasing appreciation for the importance of indication in post-surgical outcomes, especially THA for hip fracture compared to elective THA [30]. Other recent studies using the NSIQP database have implicated longer-term measures of renal function, including estimated glomerular filtration rate (eGFR) as a proposed risk-stratification instrument after primary THA [31]. Similarly, eGFR has also been implicated as a marker for complications after revision TKA [32].

There were several limitations while conducting analysis for this study. To begin with, the reliability of findings obtained from large databases is often called into question. With regards to THAs in particular, a recent study demonstrated that when comparing the National Hospital Discharge Survey (NHDS) database to the Nationwide Inpatient Sample (NIS) database, over half of the factors in demographics, comorbidities, and complications following THA differed significantly between the two databases [33]. Despite this, NSQIP data are chart-abstracted and undergo routine auditing, ultimately leading to its acceptance as the standard for database studies [34]. Another limitation to this study is the means by which hydration status was determined. Because the preoperative laboratory tests were often drawn outside of the 24 h before surgery, there was potential for patients’ hydration status to be corrected prior to surgery, and the observed outcomes were due to factors other than dehydration. Additionally, although BUN/Cr ratio is a validated metric of volume status in the literature [35], this definition of dehydration status may have contributed to an overestimation of the number of patients who were dehydrated prior to surgery. Finally, though BUN/Cr ratio is traditionally used as a marker of dehydration, it may also rise for other reasons. For example, urea may also rise in hypercatabolic states, with an upper gastrointestinal bleeding, and with high-dose glucocorticoid administration. Additionally, many patients undergoing THA and TKA for osteoarthritis are taking non-steroidal anti-inflammatory (NSAID) medications which can increase BUN and creatinine levels [36]. Because of this, it could be worth examining dehydration in future studies by using alternative metrics such as orthostasis or urine specific gravity.

Despite these limitations, this study of 188,629 patients undergoing THA demonstrated that dehydration status carries no independent risk of any postoperative complications and may correlate with a small but statistically significant reduction in extended length of hospital stay when compared to patients who met laboratory criteria for preoperative hydration. This study also demonstrated similar findings in 332,485 patients undergoing TKA. When laboratory values were limited to only those drawn just before surgery, there was no reduction in risk of extended LOS or readmission and dehydration did not increase risk of complications. Future studies should be conducted to characterize dehydration by alternative metrics that more closely assess hydration status, measuring hydration status at a time closer to surgery, and examining the role of hydration in different orthopedic procedures that may be more sensitive to preoperative hydration status.

## Conclusions

In conclusion, this study found that dehydration was not independently associated with an increase in complications within the 30-day postoperative period after primary total joint arthroplasty, and did not result in an increased risk of blood product transfusion. Accordingly, this study suggests that hydration status, at least determined by the BUN and creatinine ratio, warrants further investigation in assessing a patient’s risk of complications and optimizing the postoperative recovery. Ultimately, the findings of this study can help to inform health-care providers on interpreting perioperative hydration indicators and its role in the management of patients to reduce the risk of postoperative complications.

## Supplementary Information

**Additional file 1: Supplemental Table 1.** Demographics and comorbidities for patients undergoing primary THA within 24 hours after blood BUN and Creatinine values were collected. **Supplemental Table 2.** Relative risk of complications in the 30-day postoperative period for patients undergoing primary THA within 24 hours after blood BUN and Creatinine values were collected. **Supplemental Table 3.** Demographics and comorbidities for patients undergoing primary TKA within 24 hours after blood BUN and Creatinine values were collected. **Supplemental Table 4.** Relative risk of complications in the 30-day postoperative period for patients undergoing primary TKA within 24 hours after blood BUN and Creatinine values were collected. .

## Data Availability

Data from the National Surgical Quality Improvement Program Participant Use Files for yers 2012–2019 are freely available to participating institutions. The American College of Surgeons National Surgical Quality Improvement Program and the hospitals participating in the ACS NSQIP are the source of the data used herein and they have not verified and are not responsible for the statistical validity of the data analysis or the conclusions derived by the authors.

## Abbreviations

NSQIP: National Surgical Quality Improvement Program
THA: Total hip arthroplasty
TKA: Total knee arthroplasty
ASA: American Society of Anesthesiologists
BUN: Blood urea nitrogen (mg/dL)
Cr: Creatinine (mg/dL)
BMI: Body mass index
CHF: Congestive heart failure
