# Supplementary material for: Does dehydration prior to primary total joint arthroplasty increase risk of perioperative complications?
Source: Arthroplasty. 2021 Oct 4;3:34. doi: 10.1186/s42836-021-00090-8 (PMC8796573; doi:10.1186/s42836-021-00090-8)
Supplement: Supplementary file 1 — Additional file 1: Supplemental Table 1. Demographics and comorbidities for patients undergoing primary THA within 24 hours after blood BUN and Creatinine values were collected. Supplemental Table 2. Relative risk of complications in the 30-day postoperative period for patients undergoing primary THA within 24 hours after blood BUN and Creatinine values were collected. Supplemental Table 3. Demographics and comorbidities for patients undergoing primary TKA within 24 hours after blood BUN and Creatinine values were collected. Supplemental Table 4. Relative risk of complications in the 30-day postoperative period for patients undergoing primary TKA within 24 hours after blood BUN and Creatinine values were collected. . [file 42836_2021_90_MOESM1_ESM.docx]

**^Supplemental Materials^**

**Supplemental Table 1.** Demographics and comorbidities for patients undergoing THA.

| **Demographics and Comorbidities** | **Hydration Status** | | | | ***P*^a^** |
| --- | --- | --- | --- | --- | --- |
|  | **Non-Dehydrated** | | **Dehydrated** | |  |
|  | ***n* = 5,034** | | ***n* = 3,685** | |  |
| Age (years) mean ± SD | 64.0 ± 11.7 | | 68.1 ± 10.8 | | **<0.001** |
| Sex |  |  |  |  | **<0.001** |
| Female | 2241 | 44.5% | 2480 | 67.3% |  |
| BMI (kg/m^2^) |  | |  | | **<0.001** |
| <18 | 21 | 0.4% | 39 | 1.1% |  |
| 18—25 | 936 | 18.6% | 783 | 21.3% |  |
| 25—30 | 1665 | 33.1% | 1,155 | 31.3% |  |
| 30—35 | 1401 | 27.8% | 888 | 24.1% |  |
| 35—40 | 676 | 13.4% | 496 | 13.5% |  |
| > 40 | 335 | 6.7% | 324 | 8.8% |  |
| Functional status prior to surgery |  |  |  |  | 0.133 |
| Independent | 4894 | 97.2% | 3562 | 96.7% |  |
| Dependent | 140 | 2.8% | 123 | 3.3% |  |
| ASA |  | |  | | **0.036** |
| 1 | 194 | 3.9% | 108 | 2.9% |  |
| 2 | 2,395 | 47.6% | 1,707 | 46.3% |  |
| . 3 | 2,237 | 44.4% | 1,723 | 46.8% |  |
| . ≥ 4 | 208 | 4.1% | 147 | 4.0% |  |
| Diabetes Mellitus |  | |  |  | **<0.001** |
| No diabetes mellitus | 4387 | 87.1% | 3,074 | 83.4% |  |
| Non-insulin-dependent diabetes mellitus | 472 | 9.4% | 423 | 11.5% |  |
| Insulin-dependent diabetes mellitus | 175 | 3.5% | 188 | 5.1% |  |
| Smoker |  |  |  |  | **<0.001** |
| . No | 4288 | 85.2% | 3328 | 90.3% |  |
| . Yes | 746 | 14.8% | 357 | 9.7% |  |
| Congestive Heart Failure |  |  |  |  | 0.175 |
| No | 5004 | 99.4% | 3654 | 99.2% |  |
| Yes | 30 | 0.6% | 31 | 0.8% |  |

^a^Statistical significance set at *P* <0.05. **Bold fonts** indicates statistical significance.

**Supplemental Table 2.** Relative risk of Complications in the 30-day postoperative period for patients undergoing primary total hip arthroplasty.

| **Total** | **RR^a^** | **99.7% CI** | ***P*^b^** |
| --- | --- | --- | --- |
|  |  |  |  |
|  |  |  |  |
| Any Complication | 1.06 | 0.78—1.43 | 0.594 |
| Extended length of stay (>3 days) | 0.98 | 0.85—1.13 | 0.682 |
| Blood transfusion | 1.03 | 0.83—1.27 | 0.714 |
| Readmission | 1.11 | 0.80—1.55 | 0.333 |
| Death | 0.94 | 0.17—5.10 | 0.913 |
| Urinary tract infection | 1.24 | 0.65—2.33 | 0.322 |
| Pneumonia | 0.87 | 0.35—2.20 | 0.666 |
| Return to the operating room | 1.22 | 0.79—1.88 | 0.181 |
| Wound complications | 1.60 | 0.91—2.83 | 0.014 |
| Thromboembolic complications**^c^** | 0.79 | 0.32—1.96 | 0.439 |
| Sepsis/septic shock | 1.03 | 0.36—2.93 | 0.930 |
| Respiratory complications | 0.89 | 0.18—4.44 | 0.836 |
| Cardiac complications | 0.49 | 0.15—1.56 | 0.067 |
| Renal complications | 0.90 | 0.19—4.23 | 0.838 |
| Cerebrovascular accident | 0.61 | 0.11—3.46 | 0.393 |

^a^Poisson regressions with robust error variance were used to compare these variables. Adjusted for age, gender, BMI, ASA class, diabetes, and smoking status. ^b^Bold fonts indicates statistical significance (significant at *P* <0.003 due to Bonferroni correction for multiple comparisons). RR - relative risk. CI - confidence interval (99.7% CI due to Bonferroni). ^c^includes deep vein thrombosis and pulmonary embolim.

|  |
| --- |

**Supplemental Table 3.** Demographics and comorbidities for patients undergoing primary total knee arthroplasty.

| **Total** | **Hydration Status** | | | |  |
| --- | --- | --- | --- | --- | --- |
|  | **Non-Dehydrated** | | **Dehydrated** | |  |
|  | **N = 8393** | | **N = 6345** | | **p-value^a^** |
| Age (years) mean ± SD | 65.8 ± 9.8 | | 68.3 ± 9.2 | | **<0.001** |
| Sex |  |  |  |  | **<0.001** |
| Male | 4049 | 48.2% | 1874 | 29.5% |  |
| BMI (kg/m^2^) |  | |  | | **0.001** |
| <18 | 56 | 0.7% | 56 | 0.9% |  |
| 18—25 | 728 | 8.7% | 657 | 10.4% |  |
| 25—30 | 2264 | 27.0% | 1658 | 26.1% |  |
| 30—35 | 2459 | 29.3% | 1802 | 28.4% |  |
| 35—40 | 1697 | 20.2% | 1211 | 19.1% |  |
| > 40 | 1189 | 14.2% | 961 | 15.2% |  |
| Functional status prior to surgery |  |  |  |  | **0.003** |
| Independent | 8186 | 98.1% | 6,153 | 97.4% |  |
| Dependent | 158 | 1.9% | 165 | 2.6% |  |
| ASA |  | |  | | 0.959 |
| 1 | 139 | 1.7% | 108 | 1.7% |  |
| 2 | 3613 | 43.1% | 2755 | 43.5% |  |
| . 3 | 4394 | 52.4% | 3293 | 52.0% |  |
| . ≥ 4 | 238 | 2.8% | 181 | 2.9% |  |
| Diabetes Mellitus |  | |  |  | **0.002** |
| No diabetes mellitus | 6647 | 79.2% | 4884 | 76.9% |  |
| Non-insulin-dependent diabetes mellitus | 1305 | 15.6% | 1057 | 16.7% |  |
| Insulin-dependent diabetes mellitus | 441 | 5.2% | 404 | 6.4% |  |
| Smoker |  |  |  |  | **<0.001** |
| . No | 7533 | 89.7% | 5940 | 93.6% |  |
| . Yes | 860 | 10.3% | 405 | 6.4% |  |
| CHF |  |  |  |  | 0.168 |
| No | 8345 | 99.4% | 6297 | 99.2% |  |
| Yes | 48 | 0.6% | 48 | 0.8% |  |

^a^Statistical significance set at *P* <0.05. Bolding indicates statistical significance.

**Supplemental Table 4.** Relative risk of complications in the 30-day postoperative period for patients undergoing primary total knee arthroplasty.

| **Adverse Event** | **RR^a^** | **99.7% CI** | **p-value^b^** |
| --- | --- | --- | --- |
|  |  |  |  |
| Any Complication | 0.98 | 0.90—1.07 | 0.494 |
| Urinary tract infection | 0.94 | 0.58—1.54 | 0.713 |
| Blood transfusion | 1.14 | 0.92—1.41 | 0.068 |
| Extended length of stay (>3 days) | 0.97 | 0.88—1.08 | 0.386 |
| Readmission | 1.11 | 0.86—1.44 | 0.222 |
| Death | 1.60 | 0.42—6.12 | 0.297 |
| Pneumonia | 0.88 | 0.38—2.05 | 0.655 |
| Return to the operating room | 1.31 | 0.84—2.05 | 0.070 |
| Wound complications | 1.17 | 0.73—1.88 | 0.320 |
| Thromboembolic complications^c^ | 0.95 | 0.60—1.51 | 0.731 |
| Sepsis/septic shock | 0.99 | 0.39—2.48 | 0.771 |
| Respiratory complications | 1.24 | 0.43—3.26 | 0.547 |
| Cardiac complications | 0.98 | 0.40—2.42 | 0.951 |
| Renal complications | 1.08 | 0.36—3.26 | 0.839 |
| Stroke/cerebrovascular accident | 0.55 | 0.09—3.39 | 0.333 |

^a^Poisson regressions with robust error variance were used to compare these variables. Adjusted for age, gender, BMI, functional status, diabetes, and smoking status. ^b^Bolding indicates statistical significance (significant **at *P* <0.003** due to Bonferroni correction for multiple comparisons). RR - relative risk. CI - confidence interval (99.70% CI due to Bonferroni). ^c^includes deep vein thrombosis and pulmonary embolism.
